# Molecular Characterization of a Novel Bovine Viral Diarrhea Virus Isolate SD-15

**DOI:** 10.1371/journal.pone.0165044

**Published:** 2016-10-20

**Authors:** Lisai Zhu, Haibing Lu, Yufeng Cao, Xiaochun Gai, Changming Guo, Yajing Liu, Jiaxu Liu, Xinping Wang

**Affiliations:** 1 College of Veterinary Medicine at Jilin University, Changchun, China 130062; 2 Key laboratory for Zoonosis, Ministry of Education, and Institute for Zoonosis of Jilin University, Changchun, China, 130062; 3 Changchun Institute of Biological Products, Changchun, China 130062; 4 Guangdong Haid Animal Husbandry and Veterinary Institute, Guangzhou, China 511400; South China Agricultural University, CHINA

## Abstract

As one of the major pathogens, bovine viral diarrhea virus caused a significant economic loss to the livestock industry worldwide. Although BVDV infections have increasingly been reported in China in recent years, the molecular aspects of those BVDV strains were barely characterized. In this study, we reported the identification and characterization of a novel BVDV isolate designated as SD-15 from cattle, which is associated with an outbreak characterized by severe hemorrhagic and mucous diarrhea with high morbidity and mortality in Shandong, China. SD-15 was revealed to be a noncytopathic BVDV, and has a complete genomic sequence of 12,285 nucleotides that contains a large open reading frame encoding 3900 amino acids. Alignment analysis showed that SD-15 has 93.8% nucleotide sequence identity with BVDV ZM-95 isolate, a previous BVDV strain isolated from pigs manifesting clinical signs and lesions resembling to classical swine fever. Phylogenetic analysis clustered SD-15 to a BVDV-1m subgenotype. Analysis of the deduced amino acid sequence of glycoproteins revealed that E2 has several highly conserved and variable regions within BVDV-1 genotypes. An additional N-glycosylation site (240NTT) was revealed exclusively in SD-15-encoded E2 in addition to four potential glycosylation sites (Asn-X-Ser/Thr) shared by all BVDV-1 genotypes. Furthermore, unique amino acid and linear epitope mutations were revealed in SD-15-encoded E^rns^ glycoprotein compared with known BVDV-1 genotype. In conclusion, we have isolated a noncytopathic BVDV-1m strain that is associated with a disease characterized by high morbidity and mortality, revealed the complete genome sequence of the first BVDV-1m virus originated from cattle, and found a unique glycosylation site in E2 and a linear epitope mutation in E^rns^ encoded by SD-15 strain. Those results will broaden the current understanding of BVDV infection and lay a basis for future investigation on SD-15-related pathogenesis.

## Introduction

Bovine viral diarrhea virus (BVDV) is a small, enveloped virus with a single-stranded, positive-sense RNA genome. Together with classical swine fever virus (CSFV) and border disease virus (BDV), BVDV belongs to the genus of *pestivirus* within the family of *Flaviviridae* [1]. As one of the most important viral pathogens, BVDV causes significant economic losses to cattle industry worldwide [2–4]. In addition to cattle, BVDV also infects pigs, deer, sheep and other wild animals [5–8]. Based on the cytopathic effect (CPE) on cell culture, BVDV is divided into two biotypes, the cytopathic (CP) and noncytopathic (NCP) biotypes where CP or NCP isolates are divided into BVDV-1, BVDV-2, and atypical BVDV-3 genotypes based on viral sequence variations [9, 10]. While the epidemic isolates for BVDV mainly belong to BVDV-1, the more recent hypervirulent BVDV-2 strains have been isolated from cattle with acute diarrhea and fatal thrombocytopenia [11–13]. Genomic sequence comparisons revealed the diversity and genetic variability of BVDV strains isolated from different herds or even in the same herd [13]. Based on the genetic variability, seventeen BVDV-1 subgenotypes and four BVDV-2 subgenotypes have been reported so far [14–18].

The genome of BVDV is approximately 12.5 kb in length containing a single open reading frame (ORF) flanked by 5’-UTR and 3’-UTR [19–21]. The ORF encodes a precursor polyprotein of about 3,900 amino acids, which is subsequently processed by viral or cellular proteases into 11 or 12 individual proteins including N^pro^, C, E^rns^, E1, E2, p7, NS2/3, NS4A, NS4B, NS5A and NS5B from the N terminus to the C terminus [20, 22, 23]. The C, E^rns^, E1 and E2 are four structural proteins, and the remains are nonstructural viral proteins [23, 24]. Out of four structural proteins, E2 has a mass of 55 KDa and is classified as type I transmembrane protein, which is associated with virus entry, viral pathogenicity and immunity. E^rns^ is structural glycoprotein that possess the intrinsic ribonuclease activity involved in virus attachment and entry into target cells. Study has demonstrated that envelope proteins are involved in several biological activities through participating host–cell interactions such as receptor binding, internalization and posttranslational modifications, in most viruses, the glycosylation [25]. Glycosylation has been demonstrated to play a crucial role in biogenesis, stability, antigenicity and infectivity. Many viruses are dependent on N-linked glycosylation for vital biological functions via promoting proper folding and subsequent trafficking using host cellular chaperones and folding factors [25]. It is well-recognized that glycosylation in many enveloped viruses is important to viral infection, and alteration of glycosylation sites affects the pathogenicity and antigenicity of the viruses [26].

In China, bovine viral diarrhea-mucosal disease (BVD-MD) was first reported in 1980 on a farm where cattle were imported from Europe. The first BVDV strain named Changchun-184 (CC-184) was isolated from the same farm and classified to BVDV-1b subgenotype based on the sequence similarity [27, 28]. In 1995, a BVDV strain named ZM-95 was isolated from pigs in the Inner Mongolia autonomous region, which showed clinical signs and gross lesions similar to classical swine fever [7], thus discovering the BVDV infection in pigs in China. Sequence analysis revealed that ZM-95 belongs to BVDV-1m subgenotype [29]. During late 1990’s and early 2000’s, BVD occurred in many regions mainly due to the booming cattle industry and the circulation of live cattle across China. Analysis of the 5’-UTR sequence of BVDV strains isolated from 2005 to 2013 revealed that majority of BVDV strains belongs to BVDV-1, and BVDV-1b and BVDV-1m were the predominant subgenotype [30–35]. Recently, BVDV-2 infections have been reported in cattle and pig populations in China [36, 37]. Although BVDV infections has increasingly been reported in China recently, the majority of those reports were only focus on the genotyping using 5’-UTR sequences, thus resulting in the molecular aspects of BVDV strains largely unexplored, especially the genomic sequence of the predominated subgenotype BVDV-1m strains. To understand the origin and evolution of BVDV strain and determine the molecular characters of the BVDV strains predominantly spread in China, we collected and characterized the BVDV strains associated with severe diarrhea and mucosal diseases, and reported here the characterization of a novel BVDV genotype/subgenotype strain SD-15 that is associated with an unusual high morbidity and mortality on a cattle farm in Shandong province, China.

## Materials and Methods

### Ethics statement

All sample collection and processing from diseased or dead cattle was performed following the protocol approved by the animal ethics committee of Jilin University.

### Outbreak investigation

A disease characterized by severe diarrhea occurred on a cattle farm with 320 cattle in Shandong province after 40 cattle purchased from Inner Mongolia autonomous region were newly introduced to the original herd in early 2015. The sick cattle were observed to manifest pyrexia, anorexia in early days, and later oral mucous ulcer and severe diarrhea containing mucous and hemorrhage excretions were found. Approximately 67% (214/320) of cattle showed clinical signs and 60% (128/214) of the sick cattle succumbed to death within 10–15 days. Treatment of the sick cattle with gentamycin or tobramycin yielded no effects on relieving the clinical signs. Spleen samples were collected from four cattle for virus isolation after postmortem examination following the protocol approved by the animal ethics committee of Jilin University, and processed for electron microscope examination and virus detection.

### Cell culture and virus isolation

Madin-Darby Bovine Kidney (MDBK) cells were grown in Dulbecco’s modified Eagle medium (DEME) containing 10% fetal calf serum (FBS) (HyClone, Logan, UT) and maintained in DMEM containing 2% FBS. For virus isolation, spleen samples were homogenized in a dilution of 1:10 (W/V) with 10 mM phosphate buffered saline (PBS), centrifuged at 10,000 × g at 4°C for 10 min, then passed through 0.45 nm filter before infecting cells. After infection, cell cultures were examined for the presence or absence of cytopathic effect (CPE) before they were frozen and thawed. The 5^th^ passages of infected cells were used as stock for further characterization.

### Indirect immunofluorescence assay (IFA)

MDBK cells were seeded into 24-well plates and infected with the 5^th^ passage of isolated virus. The uninfected MDBK cells were used as the negative controls. 24–48 h postinfection, cells were fixed with methanol/acetone (1:1) for 30 min at -20°C, blocked using 1% bovine serum albumin, and incubated with polyclonal antibody against BVDV E2 protein for 1h at 37°C before the addition of Rhodamine-conjugated goat anti-rabbit antibody (1:500 dilution). After incubation for 45 min at 37°C, the cells were washed 3 times with PBS, sealed with glycerol, and examined using the fluorescence microscope.

### RNA isolation and RT-PCR

Total RNAs were extracted from either the spleen samples or infected cell cultures using TRNzol kit (Tiangen, Beijing) following manufacturer`s instructions. Briefly, the samples were lysed in TRNzol^®^ reagent, mixed with 0.2 volume of chloroform and shaken vigorously for 30 sec. After centrifugation at 12,000 x g for 20 min at 4°C, the aqueous phase was mixed with equal volume of isopropanol and centrifuged at 12,000 x g for 20 min at 4°C. The pellets were washed with 70% ethanol and dissolved in DEPC-treated H_2_O. The resultant RNAs were kept at –80°C for further analysis.

Reverse transcriptase reactions were performed using SuperScript^TM^ II Reverse Transcriptase (Invitrogen, Carlsbad, CA). Briefly, cDNA was synthesized in a volume of 20 μl containing 25 mM Tris-HCI, pH 8.3, 37.5 mM KCI, 1.5 mM MgCI_2_, 5mM DTT, 0.25 mM each of dATP, dCTP, dGTP and dTTP, 40 units of RNase inhibitor, 200 units of M-MLV reverse transcriptase, 2 μg of total RNA, and 2.5 μM random primers. The cDNA synthesis was performed at 42°C for 60 min. PCR amplification was done using Taq DNA polymerase (Takara, Dalian, Liaoning). The reaction was performed in a total volume of 50 μl containing 20 mM Tris-HCI, pH 8.4, 50 mM KCI, 3 mM MgCI_2_, 0.25 mM each of dATP, dCTP, dGTP and dTTP, 5 unit of Taq DNA polymerase, 1 μM of each primer, and 2 μl of the cDNA synthesized above. The amplification was done after the conditions were optimized. The primers used for detection of the potential pathogens were listed as Table 1. The primers used to amplify the complete genomic sequence for SD-15 were designed based on the nucleotide sequence alignment of the known BVDV strains in the GenBank and listed as Table 2.

**Table 1:** Primers used for detection of potential agents.

Virus		Primer sequence (5’-3’)	Positions
BMCFV	S	ATGACAGCAAGAGAATTAAACT	49–70
	AS	ATGAATGACACCTCCAACAAGA	479–458
RPV		GGGCTCTCATCAGCATCTTATC	319–340
	AS	GTATTTCACCCACCTCCGTAAC	704–683
BTV	S	ATGAGATGTTTTTCATGTGTCT	193–214
	AS	TTTAGTATTGCCGTTCTTAGTC	877–856
BVDV	S	GAACCAGTTTATGACAAGGAAG	454–475
	AS	GGGCAGTCTAGTCTGTTGTGGA	875–854

BMCFV: bovine malignant catarrhal fever virus; RPV: rinderpest virus; BTV: bluetongue virus; BVDV: bovine viral diarrhea virus; S: sense; AS: antisense.

**Table 2:** Primers used for amplification of the complete genome sequence.

Primers		primer sequence(5’-3’)	Positions
P1	S	ACATGGGGTATACGAGATTTA	1–21
	AS	CTCTTGTGTGGGAGCTTTA	542–524
P2	S	CCCACTGTGTTGCTACTAAAAAT	350–372
	AS	CATCACTACCGGTAACTCTCCCAA	705–682
P3	S	TCCAGTTTACCACAGAGCCCCA	675–696
	AS	GGTCTTCTCACTTGCATCCATCATA	1337–1313
P4	S	AGAACATAACACAATGGAAC	1147–1166
	AS	AGTTTTCCAGTTTCTTTCCTAG	1841–1821
P5	S	ACTGCTCATTATCTAGTTGATGGAG	1677–1701
	AS	ACCACCTTTACTAGACACACTAGGA	2353–2329
P6	S	TTTGAGGGCACTTAGAGACTTA	2273–2294
	AS	GTCAGCAAGTTGCCCATCAT	3583–3564
P7	S	AACAGCGTGCACTATCAATTAC	3290–3311
	AS	TACCTAAAGTAGTCTGTCACATAAC	3979–3955
P8	S	ATTGTAATAGGACTAATCGTGG	3807–3828
	AS	TACCATGAGCATTTTTACTTTTG	5051–5029
P9	S	AGCCAGTACATTGAATAAAAACAGG	4760–4784
	AS	ACTAGCATGTTACCCTTCATCTCC	6313–6290
P10	S	TTTATAGCCCCTGAAGTGATGAAAG	6210–6234
	AS	ACTGCAACTTGTCTGATGTGGTC	7678–7656
P11	S	ACAGCACTCTACAAGAGCATAGC	7557–7579
	AS	CTGTGTACCAGTTCAATCAACCT	9040–9018
P12-Out	S	ACCAGGTTGGCTAAGAGATATACC	8838–8861
	AS	ATCTATCTTGTCCCTGATTGCCTC	10397–10374
P12-In	S	CAATCACCGTGATCTAGTAGAGAGG	8900–8924
	AS	CTTTTCCAGTCCTATGCCTGC	10331–10311
P13	S	ATACTCAACCCTGGGAAGTTGTC	10218–10240
	AS	CCCAAGTGTAGATAGGCTCAGGTTT	11738–11714
P14	S	GGACCCAATAGGGGCATA	11630–11647
	AS	GGGGGCTGTTAAGGGTCTT	12220–12202

Out: outer pair of primers; IN: inner pair of primers: sense; AS: antisense.

### Cloning and sequencing

PCR-amplified fragments were either directly sequenced or cloned into pGM-T vector (Tiangen, Beijing) before being sequenced by Sangon Biotech Company (Shanghai, China). Every clone was sequenced twice using T7 primers. Complete genome sequence for SD-15 isolate was obtained by joining the fragment sequences obtained above.

### Sequence analysis

Sequence analysis was performed using Lasergene 7 software (Madison, WI USA). Alignment analysis of the nucleotide sequence for SD-15 isolate with known BVDV strains was performed by CLUSTAL W algorithm [38]. Phylogenetic trees were constructed based on either the 5’-UTR sequence or the complete genome sequence using neighbor-joining method [39]. Sequence identity of SD-15 to pestiviruses were analyzed and calculated by DNASTAR software [40]. Different genotypes of pestivirus strains were randomly selected from Table 3 and used for multiple alignment analysis. Analyses of the deduced amino acid sequences for E2 and E^rns^ were performed using CLUSTAL W algorithm [38]. The potential glycosylation sites within the E2 were analyzed by the NetNGlyc 1.0 Server (http://www.cbs.dtu.dk/services/NetNGlyc).

**Table 3:** The pestivirus strain used for alignment and phylogenetic analysis.

Stains	Countries	Accession No	Collection date	Subtype	Host
GS5	China	KJ541471	2013	1a	Bovine
Singer	Argentina	DQ088995	2007	1a	-
Oregon C24V	England	AF091605	1998	1a	-
SD1	USA	M96751	-	1a	-
NADL	USA	M31182	1988	1a	-
Egy/Ismailia/2014	Egypt	KR029825	2014	1b	Bovine
12F004	South Korea	KC963967	2012	1b	Cattle
CP7	Germany	U63479	1987	1b	Cattle
AU526	USA	KF835697	2013	1b	Buffy goat
CC13B	China	KF772785	2013	1b	Cattle
Bega-like	Australia	KF896608	2012	1c	Bovine
10JJ-SKR	South Korea	KC757383	2010	1d	Cattle
Carlito	Switzerland	KP313732	2014	1e	Cattle
Suwancp	Switzerland	KC853440	1993	1k	Bovine
Suwacp	Switzerland	KC853441	1993	1k	Bovine
KS86-1cp	Japan	AB078952	-	1J	Bovine
KS86-1ncp	Japan	AB078950	-	1J	Bovine
ZM-95	China	AF526381	1995	1m	Swine
SD15	China	KR866116	2015	1m	Bovine
Camel-6	China	KC695810	2013	1q	Camelus bactrianus
SD0803	China	JN400273	2008	1q	Pig
SH-28	China	HQ258810	2009	2a	Pig
HLJ-10	China	JF714967	2011	2a	Cattle
JZ05-1	China	GQ888686	2005	2a	Cattle
XJ-04	China	FJ527854	2004	2a	Cattle
Hokudai-Lab/09	Japan	AB567658	2010	2b	Bovine
SD1301	China	KJ000672	2012	2b	Cattle
NRW19-13-8_Dup(+)	Germany	HG426490	2013	2c	Bos Taurus
Potsdam 1600	Germany	HG426491	2000	2c	Bos Taurus
SH2210-23	Germany	HG426494	2010	2c	Bos Taurus
VOE 4407	Germany	HG426495	2007	2c	Bos Taurus
BD31	USA	U70263	-	BDV	Lamb
X818	Germany	AF037405	-	BDV	Sheep
Gifhorn	Germany	KF925348	2000	BDV	Pig
H2121	Germany	GU270877	2002	BDV	Rupicapra rupicapra
Aveyron	Germany	KF918753	1984	BDV	Sheep
Brescia	Switzerland	AF091661	-	CSFV-1	-
cF114	China	AF333000	-	CSFV-1	-
JL1(06)	China	EU497410	2006	CSFV-1	Swine
Shimen	China	AF092448	-	CSFV-1	-
Heb52010	China	JQ268754	2010	CSFV-2	Swine
Alfort/Tuebingen	Germany	J04358	-	CSFV-2	-
HEBZ	China	GU592790	2009	CSFV-2	Swine
JSZL	China	KT119352	2014	CSFV-2	Pig
PC11WB	South Korea	KC149991	2011	CSFV-2	Wild boar
Zj0801	China	FJ529205	2008	CSFV-2	Swine

## Results

### BVDV is the causative agents for the outbreak

Investigation on the outbreak showed that cattle purchased from Inner Mongolia autonomous region were those to first show watery diarrhea after they were transported from the Inner Mongolia to Shandong province and introduced to the cattle farm. The sick cattle were characterized with pyrexia, oral mucous ulcer and severe diarrhea with mucous and hemorrhage. Several days later, cattle in original herd began to show the clinical signs. By the end of the outbreak, approximately 67% (214/320) of cattle manifest clinical signs and 60% (128/214) of the diseased cattle succumbed to death within 10–15 days. No obvious effect was observed for the majority of sick cattle after they were treated with gentamycin or tobramycin, suggesting the outbreak is likely associated with viral agents.

**Fig 1:**
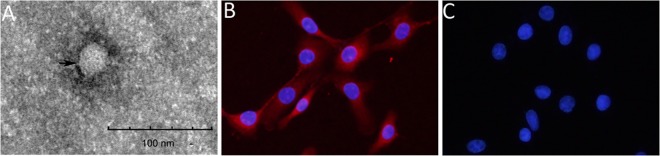
SD-15 is a noncytopathic BVDV isolate responsible for the outbreak. Viral particle was observed by electron microscopy (A). BVDV antigen was detected in MDBK cells infected by the inoculum by indirect immunofluorescence assay (B). Normal MDBK cells was detected by IFA (C). The scale bar was indicated as 100 nm.

To define the agents for outbreak, feces and spleen samples from four sick or dead cattle were collected and processed for electron microscope (EM) examination or for viral nucleic acid detection. After EM observation, viruses with a size of 30–50 nm were found in all samples (Fig 1A). As shown in Table 4, after PCR amplification, no fragments were obtained using the primers for rinderpest virus, malignant catarrhal fever virus and bluetongue virus; while fragments with expected size of 420 bp were amplified from all four cattle examined using BVDV-specific primers, suggesting that BVDV is likely the agents associated with the outbreak. Sequencing the fragments from all four diseased cattle showed they contained the same nucleotide sequences except cattle 3 has one nucleotide mutation in relation to other three cattle (not shown), further confirming that BVDV likely is responsible for this outbreak.

**Table 4:** Detection of BVDV viruses from sick cattle with RT-PCR.

Cattle No	BVDV		RPV		BTV		BMCFV	
	Spleen	Feces	Spleen	Feces	Spleen	Feces	Spleen	Feces
Calf-1	+	+	-	-	-	-	-	-
Calf-2	+	+	-	-	-	-	-	-
Calf-3	+	+	-	-	-	-	-	-
Calf-4	+	+	-	-	-	-	-	-

BVDV: bovine viral diarrhea virus; RPV: rinderpest virus; BTV: bluetongue virus; BMCFV: bovine malignant catarrhal fever virus; +: positive; -: negative

### SD-15 is a noncytopathic biotype BVDV isolate

To isolate the virus, spleen samples were processed and inoculated into MDBK cells. After 5 passages, no obvious cytopathic effects were observed for all four samples. However, the specific immunofluroscent signals were detected in the cytoplasm of infected cells using BVDV E2 antibody generated against the recombinant E2 protein (Fig 1B). No immunofluroscent signal was detected in the mock MDBK cells (Fig 1C). To assure the above results, PCRs were performed and fragments were obtained from infected cells, and revealed they were indeed BVDV-specific sequence (not shown). No fragments were amplified from normal cell controls. Those results further confirm the isolated viruses were BVDVs. The representative BVDV strain was designated as BVDV SD-15 and used for further characterization.

### Complete genome sequence of SD-15 and its encoded polyprotein

Since SD-15 was noncytopathic BVDV demonstrated to be associated with an outbreak of such an unusual high morbidity and mortality, it is necessary to unveil its complete genome sequence in an attempt to explore the molecular characters. PCR were performed and the complete genome sequence of SD-15 was obtained by joining the overlapped fragment sequences. After assembling the sequences, the complete genome of SD-15 was revealed to consist of 12285 nucleotides including a 5’-UTR of 300 nt, 3’-UTR of 200 nt, and an open reading frame (ORF) encoding a large precursor polyprotein of 3900 amino acids. Analysis of the deduced amino acid sequence showed that SD-15, like the other BVDV strains, had a conserved domain of NS3 polyprotein peptidase C31 located at 1060–1241 aa, a conserved domain of NS2 peptidase at 882–1016 aa, and the Npro endopeptidase C53 at position of 4–115 aa. Similar to the majority of BVDV-1 strains, SD-15 encoded a polyprotein putatively consisting of 4 structural proteins and 7 nonstructural proteins. No insertion/deletion or recombination was revealed between boundary of NS2 and NS3 nucleotide sequence. The complete genomic sequence of SD-15 was deposited to GenBank with an accession no KR866116.

### SD-15 belongs to BVDV-1m subgenotype

**Fig 2:**
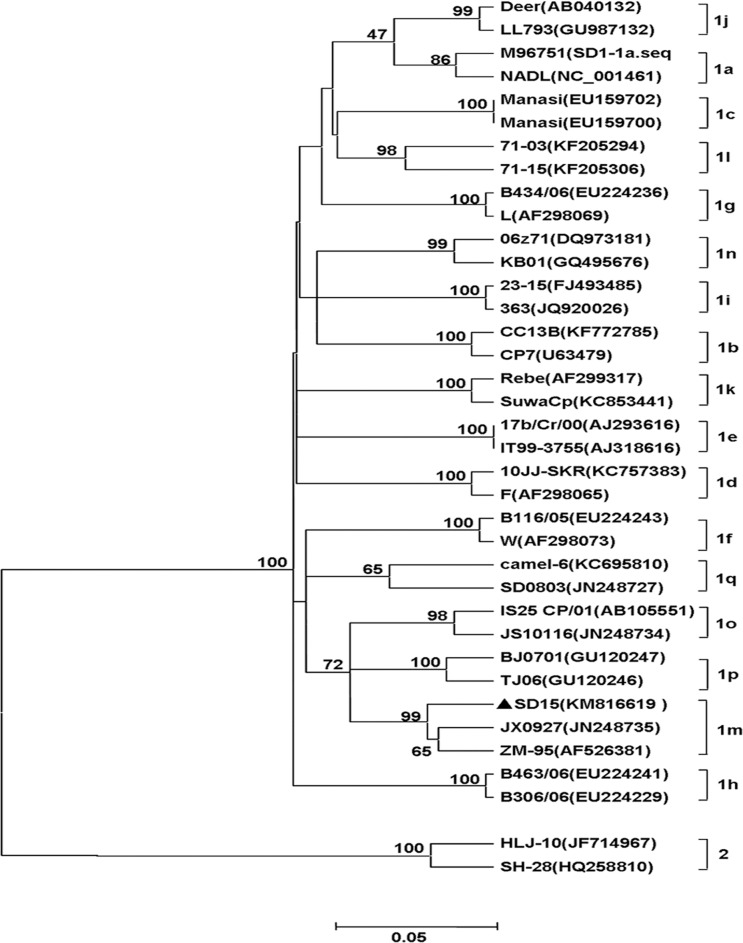
Phylogenetic analysis of SD-15 isolates based on 5’-UTR sequences. Phylogenic relationship of SD-15 to pestiviruses was generated by analyzing the 5’-UTR sequences from 36 representative BVDV isolates using neighbor-joining method. SD-15, together with BVDV ZM-95 and JX0927 was clustered to BVDV-1m subgenotype and marked as solid triangle.

**Fig 3:**
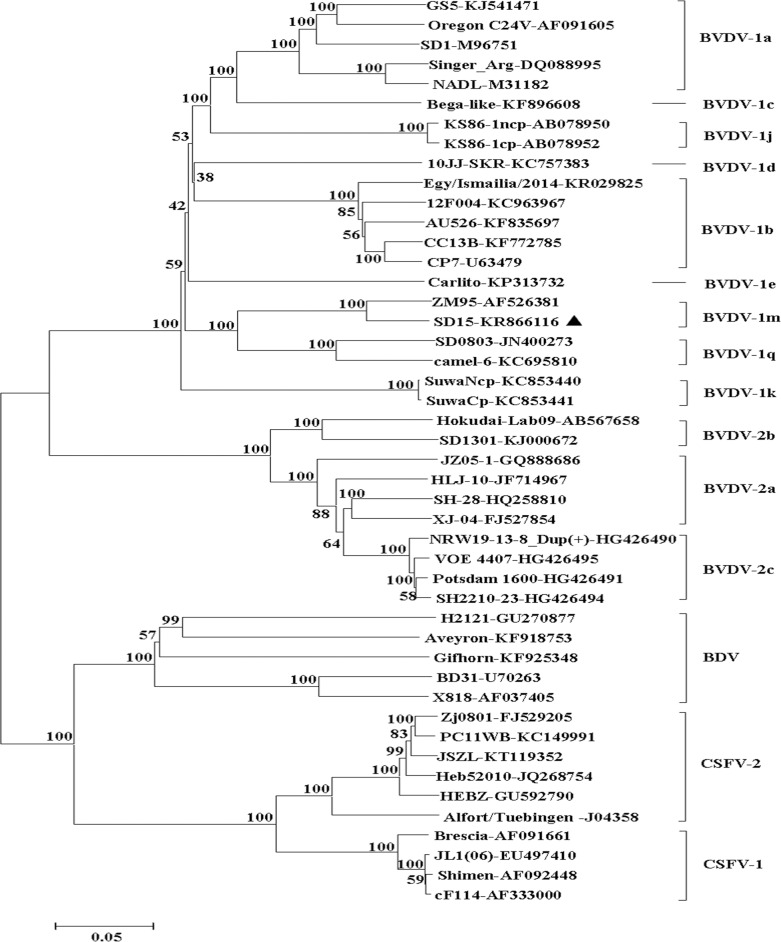
Phylogenetic analysis of SD-15 with pestiviruses based on the complete genomic sequences. The complete genome sequence of SD-15 was aligned with representative pestiviruses and phylogenetic tree was generated using the neighboring-joining methods by MEGA 5.2 software. Four major clusters including CSFV, BDV, BVDV-2 and BVDV-1 with different subgenotypes were formed and indicated, respectively. SD-15 together with ZM-95 was grouped in BVDV-1m subgenotype.

To determine the genotype of SD-15, phylogenetic analysis using neighbor-joining method was performed by analyzing the 5’-UTR sequence of SD-15 with the corresponding sequences of the 36 representative BVDV. As shown in Fig 2, the SD-15, together with ZM-95 and JX0927 was clustered to BVDV-1m subgenotype. Similar result was obtained after analysis of the complete genome sequence of SD-15 with 46 representative pestiviruses listed in Table 3, where four major distinct clusters including BVDV-1, BVDV-2, BDV and CSFV were divided, and SD-15 was grouped to a BVDV-1m subgenotype with ZM-95 within BVDV-1 isolates. Those findings further establish the phylogenetic status of SD-15 as BVDV-1m (Fig 3).

### SD-15 had the highest sequence identity with ZM-95 and a diversified sequence homology with other BVDV-1

**Fig 4:**
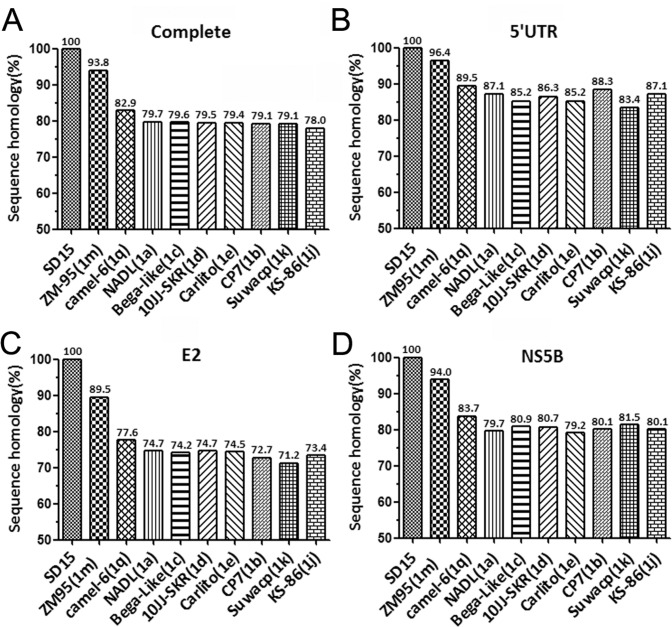
SD-15 is evolutionarily closed to ZM-95 and had diversified sequence homology with other BVDV-1 subgenotypes. The complete genome (A), 5’-UTR (B), E2 (C) and NS5B (D) nucleotide sequences of SD-15 were aligned with different BVDV-1 subgenotypes by the DNASTAR software. Sequence identities of SD-15 genes with other BVDV-1 subgenotypes were shown beyond the bars, respectively.

To determine the sequence homology of SD-15 with other BVDV-1 isolates, the complete sequence, 5’-UTR, E2 and NS5B sequences of SD-15 were aligned with corresponding sequences of BVDV-1 strains, respectively. As shown in Fig 4A, the complete genomic sequence identity of SD-15 was ranging from 78% to 93.8% with other BVDV-1 isolates, where it had the highest homology of 93.8% with ZM-95 isolate, a BVDV-1m strain originally isolated in 1995 from pigs showing clinical signs and lesions similar to classical swine fever in Inner Mongolia region. Similar patterns were also observed when 5’-UTR, E2 and NS5B genes were used for the analysis (Fig 4B–4D). Those results clearly demonstrated a closer relationship of SD-15 to the ZM-95 than other BVDV-1 strains. While the majority of SD-15-encoded genes shared higher sequence identity with ZM-95, the E2 had only 89.5% sequence homology with ZM-95 (Fig 4C), suggesting that the E2 coding region diversified more from the ZM-95 strains than other regions. It was also noted that the 5’-UTR sequence of SD-15 was relatively conserved among BVDV-1 strains in relation to other regions (Fig 4B).

### Unique glycosylation site revealed in the E2 of SD-15 strain

**Fig 5:**
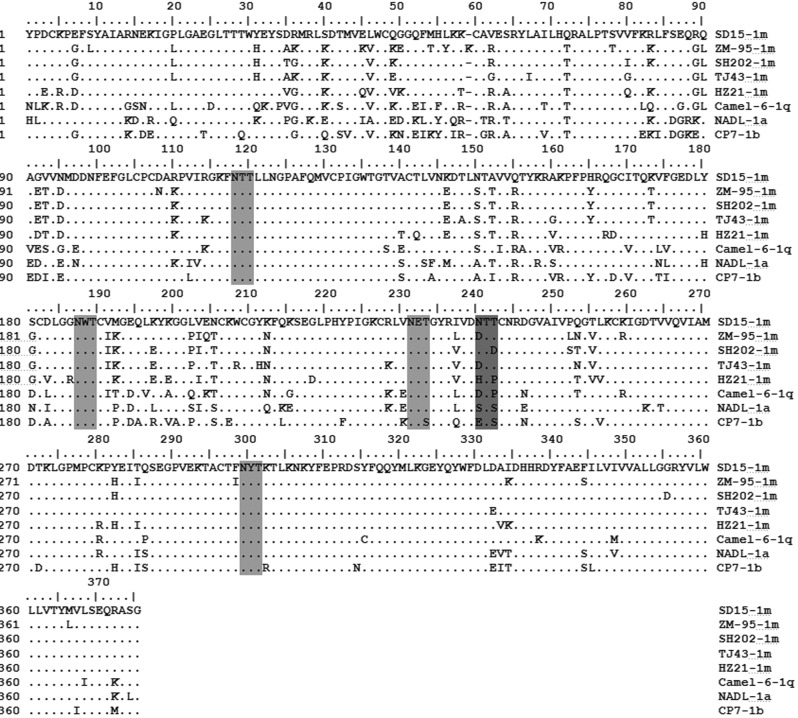
A unique glycosylation site revealed within SD-15-encoded E2 amino acid sequence. Potential glycosylation sites within E2 amino acid sequence of SD-15 were predicted using NetNGlyc 1.0 Server software. An extra and unique glycosylation site was revealed at 240NTT only in SD-15 strain in addition to four conserved glycosylation sites shared with all BVDV-1 strains examined. The glycosylation sites shared by all BVDV-1 examined were less lightly shadowed than the unique glycosylation site in SD-15.

The above results indicate that SD-15-encoded E2 was evolved faster compared with other SD-15-encoded genes. To further determine the diversity of E2 gene, alignment analysis of E2 amino acid sequences of SD-15 with representative BVDV-1 strains (1a, 1b, 1c, 1d, 1e, 1j, 1k, 1m, 1q) was performed. As illustrated in Fig 5, two highly conserved regions within the E2 sequence were revealed to locate at amino acid position of 99–136 and 286–364, respectively, indicating the important role of these conserved regions as reported previously [41]. Moreover, three variable regions were located at positions 1–63, 81–94 and 137–215 (Fig 5), suggesting those regions likely evolved faster than other regions within E2 during BVDV evolution.

To further characterize E2 protein sequence, the potential glycosylation sites were analyzed by the NetNGlyc 1.0 Server (http://www.cbs.dtu.dk/services/NetNGlyc). As shown in Fig 5, four potential glycosylation sites Asn-X-Ser/Thr were found in the E2 protein for the majority of BVDV-1 genotypes (1a, 1b, 1c, 1d, 1e, 1j, 1k, 1m, 1q), where three glycosylation sites (117 NTT,186 NWT and 298 NYT) were highly conserved and one site (230 NET) was different only in CP7 BVDV-1 strain. It is surprising to note that an additional glycosylation site (240 NTT) was found in C-terminus of E2 only in SD-15 strain. Whether this extra glycosylation site in SD-15 affects pathogenicity or antigenicity of SD-15 strain is unknown and the subject for future investigation.

### SD-15-encoded E^rns^ had a unique mutation and linear epitope

**Fig 6:**
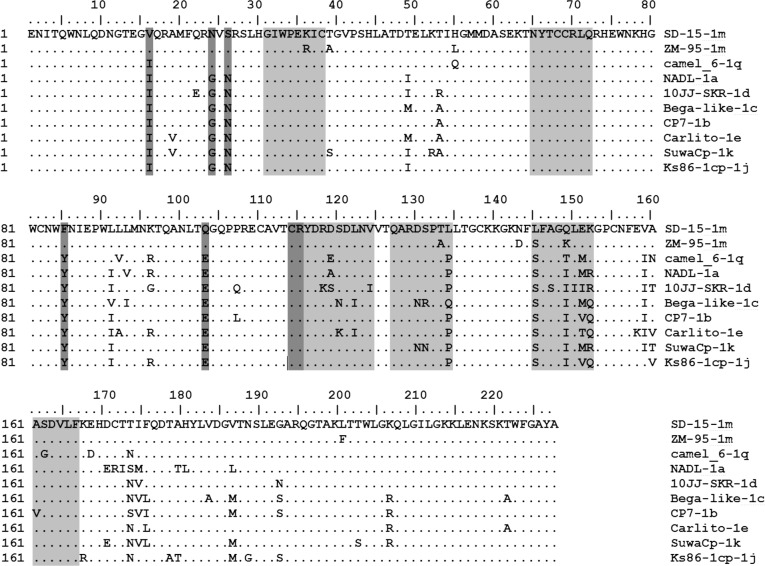
Unique amino acid and linear epitope mutations revealed in the SD-15-encoded E^rns^ protein. Analysis of the deduced amino acid sequence of SD-15-encoded E^rns^ was performed to explore the variability of linear epitopes in relation to known BVDV-1 strains. Out of seven linear epitopes of E^rns^ mapped, six were conserved among BVDV-1 genotypes and one was variable. Unique amino acid mutations and linear epitope mutations were also observed only in BVDV-1m subgenotype and highlighted.

Seven linear epitopes of E^rns^ protein were well-characterized previously [42]. They include the epitope 31GIWPEKIC38, 65NYTCCKLQ72, 127QARNRPTT134, 145SFAGTVIE152, 161VEDILY166, 114CRYDKNTDVNV124 and 116YDKNTDVNV124. Since E^rns^ is structural glycoprotein that possess the intrinsic ribonuclease activity involved in virus attachment and entry into target cells, analysis of the sequence variation will enhance the understanding of virus evolution. Therefore, diversity of E^rns^ amino acid sequence of BVDV-1 genotypes (1a, 1b, 1c, 1d, 1e, 1j, 1k, 1m, 1q) was analyzed by comparison of SD-15 with those of other BVDV strains. As shown in Fig 6, the amino acid sequence of E^rns^ of SD-15 was much conserved than E2 protein within different BVDV-1 genotypes. Seven linear epitopes were also observed in SD-15 strain, in which the linear epitopes 145SFAGTVIE152 was highly variable and the remains were conserved. The residues W33, L71, Q127, N130, S145, and G148 were conserved in all BVDV-1 strains examined except SD-15 strain, where the S145L was observed only in SD-15 isolate, suggesting the conserved function of those amino acid residues. In addition, several unique mutations including residues I16V, Y85F, E103Q in the E^rns^ of BVDV-1m, residues G24N and N26S in BVDV-1m and BVDV-1q were found.

## Discussion

BVD is one of the most significant diseases impacting the cattle industry worldwide. While reports on BVDV outbreak increased significantly in China in the last 15 years, they mainly focused on the BVDV genotyping using the 5’-UTR sequence regions [33, 43], resulting in the molecular aspects and genome sequences of BVDV related to those outbreak neglected. In this study, we characterized a novel BVDV isolate SD-15 from cattle population manifesting severe diarrhea and mucosal ulcers. Epidemiological investigation traced the cattle back to Zemeng county of Inner Mongolia autonomous region, where the first BVDV-1m strain ZM-95 was isolated. ZM-95 is a cytopathogenic BVDV strain isolated by our laboratory from pigs showing clinical signs and pathological lesions similar to classical swine fever twenty year ago in China [7]. SD-15 is a noncytopathic BVDV strain isolated in this study from cattle newly introduced to Shandong from Zemeng County of the Inner Mongolia autonomous regions. The complete genome sequence analysis and molecular characterization of SD-15 demonstrated that SD-15, together with ZM-95, belongs to BVDV-1m subgenotype that is currently reported only in China. The biotype difference between ZM-95 (CP) and SD-15 (NCP) was likely due to long-term interactive adaption of virus to host. Since BVDV-1m was exclusively reported in China, we assume that BVDV-1m had been existing in cattle population for long time and evolved to infect other species such as pigs, sheep and deer. This is evidenced by our previous etiological investigation that both cattle and sheep in the Inner Mongolia region had a very high BVDV infection [44]. The finding that the majority of BVDV-1 strains from other regions in China were BVDV-1m [33, 45], indicating BVDV-1m strains were likely to spread widely in China. Although we had no direct evidence bridging ZM-95 and SD-15 BVDV strain currently, the results that ZM-95 and SD-15 were isolated from pigs and cattle, respectively, in the same region of the Inner Mongolia indicate that BVDV-1m strain is likely the main subgenotype circulating in the Inner Mongolia. Since Inner Mongolia region was the hub of cattle trade and cattle industry in China, the BVDV-1m strains detected in other regions in China are probably originated from Inner Mongolia.

The first BVD was reported in Jilin China in 1980 [27]. Since then, BVD outbreak has not been increasingly reported until the later 1990s’ and early 2000s’. Recently, BVDs were reported in many regions across China and the predominant genotypes/subgenotypes were BVDV-1b and BVDV-1m [15, 34, 35]. Since the phylogenetic analysis clustered the first BVDV strain CC-184, which was isolated in China by our laboratory from the cows imported from Europe, to BVDV-1b [27–28], it is likely that the current BVDV-1b strains reported in China were originally from European countries [28, 46]. Majority of BVDV strains isolated recently from Jilin and other regions in China including BVDV-CC13B, JL-1 isolates were BVDV-1b subgenotype that shared the highest sequence identity with the CP7 strain originally isolated in Germany [21, 47]. Those results further support our hypothesis that predominant BVDV-1b subgenotype currently spread in China is originated from Europe.

Sequencing SD-15 genome revealed a 12,285 nucleotides that encoded a polyprotein consisting of 4 structural proteins and 7 nonstructural proteins. Like the majority of BVDV-1 strains, SD-15 contains no cellular sequence or viral sequence insertion within the boundary NS2/NS3 nonstructural proteins. Alignment analysis using the complete sequence, 5’-UTR, E2 and NS5B showed SD-15 had a sequence identity of 93.8% with ZM95, which is much higher than those of BVDV-1 strains such as BVDV-1a NADL (79.7%) and BVDV-1b CP7 (79.1%), indicating SD-15 had lower sequence homology to the BVDV-1a and BVDV-1b subgenotypes than the rest of the BVDV-1 subgenotype. It is interesting to note that SD-15-encoded E2 had the highest sequence diversity within BVDV-1 viruses, especially at the regions of amino acid residues 1–63, 81–94 and 137–215, indicating that SD-15-encoded E2 evolved faster compared with other SD-15-encoded genes during BVDV evolution.

It has been demonstrated that all three glycoproteins encoded by the pestiviruses are involved in the virus attachments and entry into the target cells [25]. The glycoprotein E2 is essential for virus entry and infectivity via the formation of E1-E2 heterodimer. E^rns^ has intrinsic ribonuclease activity that functions to inhibit the production of type I interferons and help in the development of persistent infection [25, 26]. Studies has demonstrated that variation in the amino acid and antigenic structure, disulfide bond formation, glycosylation, and RNase activity affect the virulence of pestiviruses to the animals. Also, the antigenic difference in glycosylation influence the efficacy of vaccine. As one of the most common forms of protein modifications, glycosylation play an important role in virus infection and alteration of glycosylation sites had significant impact on virus survival, pathogenicity, antigenicity, and transmissibility [25, 48–50]. The discoveries of an extra and unique glycosylation site in E2 of SD-15 strain in addition to four glycosylation sites shared by all BVDV-1 subgenotype suggest that this unique glycosylation site may have an effect on the virulence and pathogenicity of SD-15. The findings of unique amino acid mutations and linear epitope alteration in E^rns^ of SD-15 suggest that these mutations likely also affect the antigenicity and pathogenicity of the SD-15, which is the subject for future exploration.
